# Comparing participants from a randomized trial and screened non‐participants: Implications for generalizability of surgery and exercise therapy in young adults with meniscal tears

**DOI:** 10.1002/jeo2.70791

**Published:** 2026-05-29

**Authors:** Jonas Bloch Thorlund, Dorte Thalund Grønne, Per Hölmich, Martin Lind, Søren Thorgaard Skou

**Affiliations:** ^1^ Center for Muscle and Joint Health, Department of Sports Science and Clinical Biomechanics University of Southern Denmark Odense Denmark; ^2^ Research Unit for General Practice, Department of Public Health University of Southern Denmark Odense Denmark; ^3^ Department of Anaesthesiology and Intensive Care Medicine Odense University Hospital Odense Denmark; ^4^ The Research and Implementation Unit PROgrez, Department of Physiotherapy and Occupational Therapy Næstved‐Slagelse‐Ringsted Hospitals, Denmark, Region Zealand Slagelse Denmark; ^5^ Sports Orthopedic Research Center—Copenhagen (SORC‐C), Department of Orthopaedic Surgery Copenhagen University Hospital, Amager‐Hvidovre Copenhagen Denmark; ^6^ Department of Orthopedics Aarhus University Hospital Aarhus Denmark

**Keywords:** arthroscopy, exercise therapy, knee, meniscus, RCT

## Abstract

**Purpose:**

To investigate the generalizability of the study population and treatment effects observed in a randomized controlled trial comparing a strategy of early surgery to a strategy of exercise therapy and patient education (with the option of later surgery) for young adults with meniscal tears, through comparison with a parallel clinical cohort.

**Methods:**

We compared patient characteristics of patients (*n* = 121) from the Danish RCT on Exercise versus Arthroscopic Meniscal Surgery for Young Adults (DREAM) to patients (*n* = 182) aged 18–40 years with a meniscal tear participating in a parallel clinical cohort (i.e., those who were screened for the RCT, but not participating for various reasons), to assess the generalizability of the DREAM study population. In addition, we compared observed treatment effects between the trial and cohort (Knee injury and Osteoarthritis Outcome Score, mean of 4/5 subscales—KOOS_4_) of surgery (trial [*n* = 60] vs. cohort [*n* = 52]) and exercise (trial [*n* = 61] vs. cohort [*n* = 51]).

**Results:**

Patients participating in the DREAM trial and the clinical cohort were, on average, similar in most patient characteristics and level of patient‐reported symptoms at baseline, except for symptom duration, symptom onset and KOOS symptom subscale scores, where statistically significant differences were observed. Similar baseline to 12‐month average KOOS_4_ trajectories were observed for trial and cohort patients following surgery (time × group *p* = 0.98; adjusted mean difference in change 0.7, 95% confidence interval [CI] –7.1 to 8.5) and exercise therapy (time × group *p* = 0.62; adjusted mean difference in change 3.2, 95% CI –4.3 to 10.7).

**Conclusions:**

Overall, both baseline characteristics and observed improvements of surgery and exercise therapy observed in the DREAM trial were largely comparable to those observed in the clinical cohort, suggesting that the DREAM trial results were generalizable to patients aged 18–40 years with meniscal tears observed in daily clinical practice.

**Level of Evidence:**

Level II.

**Abbreviations**
ADL: activities of daily living
APM: arthroscopic partial meniscectomy
BMI: body mass index
DREAM: Danish RCT on Exercise versus Arthroscopic Meniscal Suegery for Young Adults
IQR: interquartile range
KOOS: Knee injury and Osteoarthritis Outcome Score
MCID: minimal clinically important difference
MRI: magnetic resonance imaging
RCT: randomized controlled trial
SD: standard deviation
STARR: The Study of Traumatic meniscal tears: Arthroscopic Resection versus Rehabilitation
WOMET: Western Ontario Meniscal Evaluation Tool

## INTRODUCTION

A tear to the meniscus is a common knee injury in young active adults. Such tears have traditionally been treated surgically with either arthroscopic partial meniscectomy (APM) or meniscal repair to preserve as much as the meniscus as possible to reduce later osteoarthritis risk [1, 11, 13].

In 2022, two randomized controlled trials (RCTs) comparing surgical versus non‐surgical treatment strategies (with optional later surgery) for young patients with meniscal tears were published [17, 24]. Both groups showed clinically important improvements in patient‐reported outcomes, with no clinically or statistically significant between‐group differences at 1‐ and 2‐year follow‐up [3, 17, 24]. At 2 years, 30% and 41% of patients initially assigned to exercise therapy had undergone surgery [3, 24], suggesting that a strategy of ‘exercise first, maybe surgery later’ could be a sensible treatment strategy for these patients [23].

A general concern about RCTs is how well the patient population and observed treatment effects represent those in clinical practice. The patient population may, for instance, differ due to narrow in‐ and exclusion criteria, or selection bias may be an issue if patients enroled in a trial differ substantially from those declining to participate [7, 12]. Furthermore, treatments offered in trials may be delivered in more optimal and controlled settings by expert clinicians, which may lead to larger treatment effects than the average treatment effect observed in daily clinical practice [12].

To evaluate the generalizability of the patient population and the treatment effects observed in the Danish RCT on Exercise versus Arthroscopic Meniscal Surgery for Young Adults (DREAM) trial, we made a comparison to a parallel collected clinical cohort, consisting of young patients with meniscal tears screened, but not included in the RCT. We aimed to (i) compare the patient characteristics of the RCT study population to those of the clinical cohort and (ii) compare the treatment effects of both meniscal surgery and exercise therapy observed in the trial, to the improvements of the same interventions in the clinical cohort. We hypothesized that both patient characteristics and treatment responses in the RCT would be similar to those in the clinical cohort.

## METHODS

### Patients

We included patients from the original DREAM trial [17] and a parallel clinical cohort, which included patients screened for eligibility in the DREAM trial but not participating in the trial for various reasons (more details below) [18].

#### DREAM trial patients

Patients included in the DREAM trial were adults 18–40 years of age with knee pain, a clinical history and symptoms consistent with a meniscal tear, verified on magnetic resonance imaging (MRI), who were deemed eligible for meniscal surgery (APM or repair) by an orthopaedic surgeon in one of seven orthopaedic departments, were willing to be randomized and provided oral and written informed consent.

Exclusion criteria were; prior surgery on the affected knee, clinical suspicion of a displaced bucket handle tear characterized by acute knee locking or an extension deficit (confirmed on MRI), fracture of the affected extremity within the past 12 months, complete rupture of any knee ligament, participation in supervised exercise therapy within the last 3 months prior to recruitment and other reasons for exclusion (inability to understand Danish, not mentally fit to participate, congenital discoid meniscus) [17, 18].

#### Clinical cohort patients

Patients not eligible or willing to participate in the DREAM trial during the screening process were invited to participate in the parallel clinical cohort. This included patients who failed to meet the full list of eligibility criteria but met the age criteria (18–40 years) and exhibited clinical suspicion and symptoms of a meniscal tear. Additionally, patients who met the eligibility criteria but were not willing to participate (e.g., due to lack of time or not willing to be randomized) in the DREAM trial were also invited to join the cohort. Some patients underwent acute meniscal surgery (and could therefore not be randomized); these patients were also invited to participate in the cohort [18].

To be eligible for comparison of patient characteristics with the RCT population, clinical cohort patients had to fulfil additional criteria to make sure that they had a meniscal tear. Thus, clinical cohort patients were required to fulfil at least one of the following additional criteria:

- MRI verified meniscal tear (observed as part of RCT screening).
- Meniscal tear undergoing surgery.
- At least two out of four positive clinical tests at baseline, including joint line tenderness (medial or lateral), McMurray's and Thessaly's test (at least one had to be McMurray or Thessaly).

To be eligible for comparison of treatment effects, patients in the clinical cohort were also required to have had surgery or exercise therapy as treatment (self‐reported at the 3‐month follow‐up), and having Knee injury and Osteoarthritis Outcome Score (KOOS_4_) scores available on at least one follow‐up time (i.e., 3, 6 or 12 months) in addition to baseline KOOS_4_ scores.

### Interventions

#### DREAM trial

Patients in the DREAM trial were randomized (1:1 ratio) to either meniscal surgery or supervised exercise therapy and patient education (with the option of later surgery). The surgery consisted of either APM or meniscal repair at the surgeon's discretion following standard procedures [16]. After the surgery, patients undergoing APM received a leaflet with exercises to facilitate at least a minimum level of postoperative rehabilitation. Patients undergoing meniscal repair received postoperative rehabilitation, ranging from control of range of motion and instructions in standard postoperative exercises to a supervised exercise programme based on individual patients' needs [17].

Patients randomized to supervised exercise therapy and patient education participated in supervised group‐based neuromuscular and strengthening exercise therapy lasting for 12 weeks (2 weekly sessions lasting 60–90 min), and two patient education lessons, one placed at the beginning and one at the end of the exercise programme. The exercise programme was developed based on evidence from other types of knee injuries and osteoarthritis [2, 6, 10, 19, 20] and feasibility tested before the RCT in collaboration with patients and experienced physical therapists [21].

#### Clinical cohort

Non‐randomized patients in the parallel clinical cohort were treated according to usual clinical practice for young adults with meniscal tears, and grouping of patients in this study (surgery or exercise therapy) was based on self‐reported information on treatment at the 3‐month follow‐up. The decision to opt for either meniscal surgery or exercise therapy as treatment in the clinical cohort was based on the clinician's assessment and the shared decision‐making process, incorporating individual patient preferences and values. As the treatment occurred as part of normal clinical practice, treatments were not monitored.

### Outcomes

Patient‐reported outcomes were collected using online questionnaires at baseline, 3, 6 and 12 months. To ensure optimal data collection in the DREAM trial, a system was implemented to notify non‐responders by email, then SMS and finally phone calls if they had not replied to the questionnaires. Due to fewer resources for follow‐up in the cohort, these patients only received reminders by email in case of non‐response.

### Primary outcome

The primary outcome was the between‐group difference in mean improvement of the Knee injury Osteoarthritis Outcome Score (KOOS_4_) from baseline to 12‐month follow‐up, comparing meniscal surgery subgroups (trial vs. cohort) and exercise therapy subgroups (trial vs. cohort). The KOOS is a knee‐specific, valid and reliable patient‐reported outcome measure for individuals in the continuum from knee injury to osteoarthritis [5, 15, 22] and is assessed using five subscales (pain, symptoms, activity of daily living, function in sport and recreation and quality of life) all ranging from 0 to 100, with lower scores indicating worse pain, symptoms, function and quality of life. The KOOS_4_ is the average score of four of the five subscales, excluding the activities of daily living (ADL) subscale, as this construct is not sensitive in the young population [5]. Similar to the original trial, we considered a between‐group difference of 10 KOOS points as the minimal clinically important difference (MCID) [14], although acknowledging that the MCID for the KOOS score may differ between subscales and vary by population and context [8].

### Secondary outcome measurements

Secondary outcomes were differences in changes between groups from baseline to 12‐month follow‐up on the five KOOS subscale scores (each scored from 0 to 100 as the primary outcome), and the Western Ontario Meniscal Evaluation Tool (WOMET), a meniscus‐specific, valid and reliable patient‐reported outcome measure (converted to scores from 0 to 100, with lower scores indicating worse quality of life) [4, 9].

### Statistics

Descriptive data are presented as means with standard deviation (SD), medians and interquartile range (IQR) or numbers with percentages as appropriate. For comparison of patient characteristics between the trial and the clinical cohort, continuous data were compared using unpaired *t* test or Kruskal–Wallis depending on distribution, and categorical data using a *χ* ^2^ test.

The primary analysis was the between‐group differences in mean improvement of KOOS_4_ from baseline to 12‐month follow‐up, comparing meniscal surgery groups (trial vs. cohort) and exercise therapy groups (trial vs. cohort). This was analysed using a mixed linear model, which incorporated the groups (surgery trial, surgery cohort, exercise trial and exercise cohort) and time (baseline, 3‐, 6‐ and 12 months) as fixed effects and patients as random effects and the interaction between group and time (group × time) to evaluate potential differences in trajectory over time between meniscal surgery and exercise therapy subgroups, respectively. We repeated these analyses adjusted for sex, age and body mass index (BMI). All secondary outcomes were analysed using the same mixed linear model approach, including adjusted analyses, as for the primary outcome. We used STATA/BE 19.0 software (StataCorp LLC) for all analyses with a 0.05 level of significance.

## RESULTS

**Figure 1:**
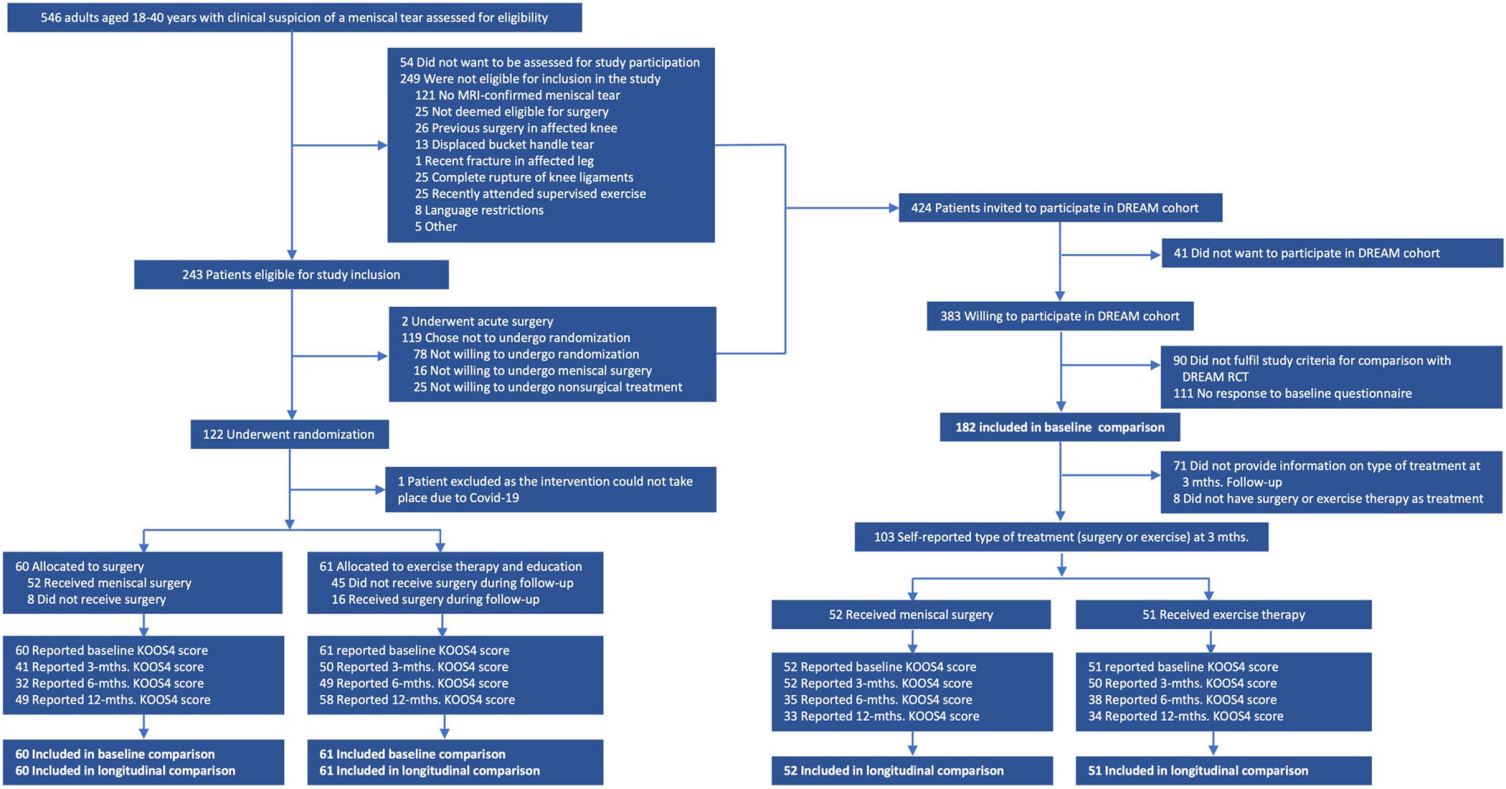
Flow of study participants in the DREAM trial and DREAM cohort. DREAM, Danish RCT on Exercise versus Arthroscopic Meniscal Surgery for Young Adults; KOOS_4_, Knee injury and Osteoarthritis Outcome Score, mean of 4/5 subscales; MRI, magnetic resonance imaging; RCT, randomized controlled trial.

From January 2017 to December 2019, 546 patients aged 18–40 years with a clinical suspicion of a meniscal tear were assessed for eligibility at the seven recruiting orthopaedic departments involved in the DREAM trial. Of these, 243 were deemed eligible, with 122 ultimately being randomized and entering the trial. Due to COVID‐19, one patient could not undergo surgery as randomized and was excluded from the trial, meaning that 60 patients were randomized to meniscal surgery and 61 were randomized to exercise therapy (with the option of later surgery). In total, 424 patients not entering the DREAM trial were invited to participate in the clinical cohort. Of these, 182 met the inclusion criteria for the baseline comparison of patient characteristics, and 103 provided information on treatment (i.e., surgery or exercise) and KOOS_4_ scores, making them eligible for the longitudinal comparison of treatment outcomes between trial patients and clinical cohort patients (Figure 1).

### Comparison of patient characteristics: Trial versus clinical cohort

Patients participating in the DREAM trial and the clinical cohort were, on average, similar in most patient characteristics and level of patient‐reported symptoms at baseline (i.e., KOOS and WOMET scores). However, KOOS symptom subscale scores were worse in patients in the clinical cohort, and cohort patients appeared to have a shorter symptom duration, and a larger proportion of patients with traumatic tears were included in the cohort compared to those entering the trial (Table 1).

**Table 1:** Comparison of baseline patient characteristics, DREAM trial versus clinical cohort.

	Trial (*n* = 121)	Cohort (*n* = 182)	*p* value
Age, mean (SD)	29.7 (6.6)	28.8 (6.4)	0.27
Females, *n* (%)	34 (28%)	64 (35%)	0.20
BMI, mean (SD)^a^	26.2 (4.5)	25.7 (4.4)	0.39
Tegner, median (IQR)^b^	5 (4–7)	6 (5–8)	0.23
Symptom duration			
0–3 months	24 (20%)	71 (40%)	
4–6 months	45 (37%)	49 (28%)	
7–12 months	24 (20%)	25 (14%)	>0.01
13–24 months	11 (9%)	19 (11%)	
>24 months	17 (14%)	14 (8%)	
Symptom onset			
Slowly evolved over time	32 (26%)	30 (17%)	
Semi‐traumatic	49 (41%)	67 (38%)	0.05
Traumatic	40 (33%)	81 (46%)	
Positive clinical tests			
Medial joint line tenderness, *n* (%)	77 (66%)	120 (72%)	0.28
Lateral joint line tenderness, *n* (%)	39 (34%)	46 (28%)	0.29
Thessaly, *n* (%)	97 (83%)	119 (76%)	0.15
McMurray, *n* (%)	83 (71%)	115 (72%)	0.80
Mechanical symptoms, *n* (%)	63 (52%)	84 (47%)	0.41
KOOS scores, mean (SD)			
KOOS_4_	55.8 (16.1)	53.7 (18.1)	0.29
Pain	66.4 (17.0)	64.7 (19.0)	0.44
Symptoms	68.8 (16.9)	64.3 (20.1)	0.04
ADL	76.5 (18.0)	74.7 (19.4)	0.41
Sport/Rec	42.4 (24.3)	38.8 (27.6)	0.26
QOL	45.8 (18.0)	46.9 (18.8)	0.61
WOMET total %score, mean (SD)	45.4 (19.6)	45.9 (18.9)	0.82

*Note*: Percentages may not add up to 100% due to rounding. Missing data: age *n* = 4; BMI *n* = 8; Tegner *n* = 4; Symptom duration *n* = 4; Symptom onset *n* = 4; Medial joint line tenderness *n* = 19; Lateral joint line tenderness *n* = 21; Thessaly *n* = 29; McMurray *n* = 27; Mechanical symtoms *n* = 4; KOOS scores *n* = 4; WOMET = 5.

Abbreviations: ADL, function during activities of daily living; BMI, body mass index; IQR, interquartile range; KOOS, Knee Injury and Osteoarthritis Outcome Score (range: 0 = worst to 100 = best); QOL, quality of life; Sport/Rec, function during sport and recreational activities; SD, standard deviation; WOMET, Western Ontario Meniscal Evaluation Tool.

^a^ BMI calculated as weight in kilograms divided by the square of the height in metres.

^b^ The Tegner Activity Scale ranges from 0 to 10, with 0 representing sick leave or disability pension because of knee problems to 10 representing competitive sports such as European football (national and international elite level).

### Comparison of outcomes after surgery: Trial versus clinical cohort

**Figure 2:**
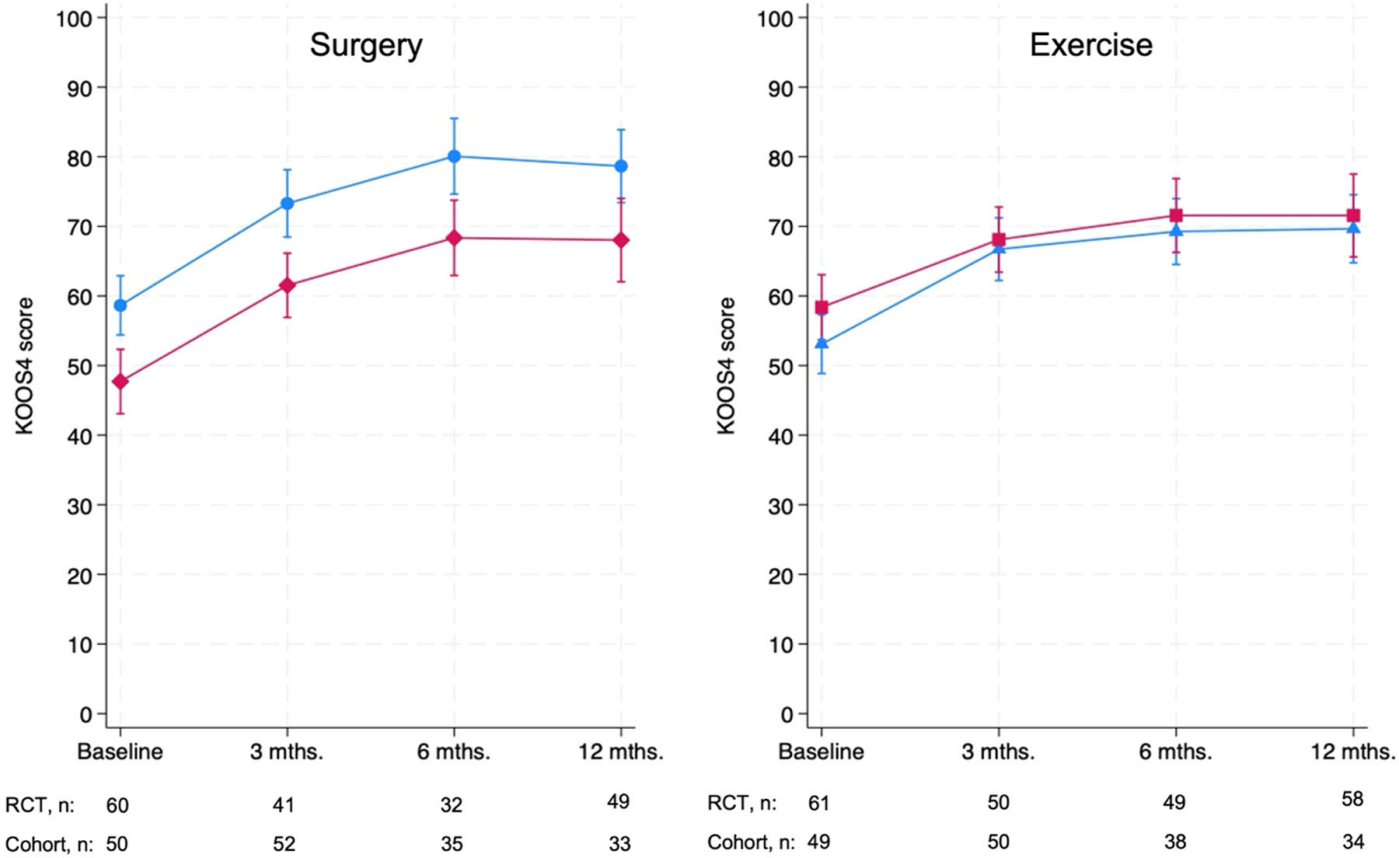
Unadjusted mean (95% CI) Knee injury and Osteoarthritis Outcomes Scores (KOOS_4_) comparing tri versus cohort patients receiving either surgery (RCT = blue; cohort = red, time × group interaction *p* = 0.98) or exercise (RCT = blue; cohort = red, time × group interaction *p* = 0.62), respectively. CI, confidence interval; RCT, randomized controlled trial.

DREAM trial patients randomized to surgery had better KOOS_4_ scores at baseline and had better KOOS_4_ scores at all time points up to 12 months compared to cohort patients having surgery (Supporting Information S1: Table 1). However, both groups improved ~20 KOOS_4_ points from baseline to 12 months with no statistically significant difference in improvement between groups (adjusted mean difference in change: 0.7, 95% confidence interval [CI] −7.1 to 8.5) (Table 2), and no difference was observed in the trajectory of KOOS_4_ over time (*p* = 0.98, Figure 2). A similar pattern of no statistically significant difference in improvement between DREAM trial versus cohort patients was observed for all KOOS subscales and WOMET (Table 2).

**Table 2:** Comparison of improvements in patient‐reported outcomes between patients having surgery in the trial versus clinical cohort and patients having exercise therapy in the trial versus clinical cohort, respectively.

	Mean improvement in surgery		Difference in improvement in surgery, trial versus cohort		Mean improvement in exercise therapy		Difference in improvement in exercise therapy, trial versus cohort	
	Trial	Cohort	Crude	Adjusted^a^	Trial	Cohort	Crude	Adjusted^a^
KOOS_4_ b	20.0 (15.0 to 25.0)	20.3 (14.5 to 26.1)	−0.3 (−8.0 to 7.4)	0.7 (−7.1 to 8.5)	16.6 (11.9 to 21.2)	13.2 (7.4 to 18.9)	3.4 (−4.0 to 10.8)	3.2 (−4.3 to 10.7)
KOOS subscales^b^								
Pain	16.6 (11.6 to 21.6)	19.2 (13.4 to 25.0)	−2.6 (−10.2 to 5.1)	−1.5 (−9.4 to 6.3)	13.6 (8.9 to 18.3)	11.0 (5.2 to 16.7)	2.7 (−4.8 to 10.1)	2.7 (−4.8 to 10.3)
Symptoms	14.9 (9.8 to 20.0)	18.6 (12.7 to 24.6)	−3.7 (−11.6 to 4.1)	−3.1 (−11.2 to 5.0)	11.2 (6.3 to 16.0)	12.4 (6.5 to 18.4	−1.3 (−8.9 to 6.4)	−1.4 (−9.2 to 6.4)
ADL	12.9 (8.4 to 17.4)	16.3 (11.1 to 21.6)	−3.5 (−10.4 to 3.4)	−2.3 (−9.3 to 4.7)	10.6 (6.4 to 14.8)	5.7 (0.5 to 10.9)	4.9 (−1.8 to 11.6)	4.7 (−2.1 to 11.5)
Sport/Rec	27.6 (20.1 to 35.0)	30.6 (21.8 to 39.4)	−3.0 (−14.6 to 8.5)	−0.9 (−12.7 to 10.8)	24.3 (17.3 to 31.3)	16.7 (8.0 to 25.4)	7.6 (−3.6 to 18.7)	7.5 (−3.8 to 18.8)
QOL	20.7 (14.8 to 26.6)	12.8 (5.9 to 19.6)	7.9 (−1.1 to 16.9)	8.3 (−0.9 to 17.4)	16.3 (10.8 to 21.8)	12.4 (5.6 to 19.1)	3.9 (−4.8 to 12.6)	3.4 (−5.3 to 12.2)
WOMET^c^	401 (302 to 500)	267 (159 to 374)	134 (−12 to 280)	164 (−15 to 313)	301 (211 to 390)	212 (105 to 318)	89 (−50 to 228)	84 (−56 to 225)

Abbreviations: ADL, function during activities of daily living; BMI, body mass index; KOOS, Knee Injury and Osteoarthritis Outcome Score; QOL, quality of life; Sport/Rec, function during sport and recreational activities; WOMET, Western Ontario Meniscal Evaluation Tool.

^a^ Adjusted for age, sex and BMI.

^b^ The KOOS includes subscales for pain, symptoms, function in daily living, function in sport and recreation and quality of life, with scores ranging from 0 (worst) to 100 (best). KOOS_4_ is the mean score of four of the five KOOS subscale scores (i.e., pain, symptoms, function in sport and recreation and quality of life).

^c^ WOMET results were converted to scores from 0 to 100, with lower scores indicating worse quality of life.

### Comparison of outcomes after exercise: Trial versus clinical cohort

No statistically significant difference in KOOS_4_ scores was observed between DREAM trial patients randomized to exercise and cohort patients having exercise as treatment at any time point from baseline to 12 months (Supporting Information S1: Table 1). Similarly, no difference in improvement in KOOS_4_ scores from baseline to 12 months was observed between DREAM trial and clinical cohort patients (adjusted mean difference in change: 3.2, 95% CI −4.3 to 10.7) (Table 2) or in the trajectory of KOOS_4_ scores over time (*p* = 0.62, Figure 2). A similar pattern of no statistically significant difference between DREAM trial and cohort patients was observed for all KOOS subscales and WOMET (Table 2).

## DISCUSSION

We observed that the study population in the DREAM trial was largely comparable with the population in the clinical cohort representing patients aged 18–40 years with a meniscal tear. Furthermore, improvements in patient‐reported outcomes of both surgery and exercise therapy observed in the DREAM trial were comparable to the improvements observed in patients in the clinical cohort, despite some differences in baseline symptoms between patients randomized to surgery and patients undergoing surgery in the cohort.

Investigating generalizability of results from RCTs to clinical practice is important, as trials may include selected patients due to specific eligibility criteria, and selection bias may also occur due to the inability to screen and recruit all patients in clinical practice. Furthermore, treatments may perform differently in selected patients and/or care may be optimized in the trial setting and delivered by specially trained clinicians, resulting in better outcomes than observed in routine care [12, 26].

Patients included in the DREAM trial and patients in the clinical cohort were similar at baseline in terms of age, sex, BMI, physical activity level, proportion with mechanical symptoms and patient‐reported outcomes (except for the KOOS symptom subscale). However, it appeared that a larger proportion of patients in the cohort reported shorter symptom duration and traumatic symptom onset. Speculatively, these attributes could have influenced the patient's willingness to participate in the trial and the clinician's referral to the trial, if patients/clinicians considered meniscal surgery a better or quicker ‘fix’ than exercise therapy in this situation. Similar to our results, previous research in patients with degenerative meniscal tears participating in RCTs evaluating the generalisability to the target population also observed that patients included in the RCTs were largely representative of the target population [25].

DREAM trial patients randomized to surgery and cohort patients having surgery experienced a similar trajectory of KOOS_4_ scores over time and clinically relevant improvements in KOOS_4_ scores of about 20 points from baseline to 12 months were observed in both groups. Similar improvements were observed despite cohort patients having substantially worse KOOS_4_ scores (~11 points) at baseline, which persisted over the 12 months. A higher proportion of surgically treated cohort patients had shorter symptom duration, which speculatively could be due to a higher proportion of patients with acute meniscus injury types in cohort patients and could be linked to lower preoperative KOOS scores before surgical treatment initiation. For patients treated with exercise therapy, the same pattern of no difference in change from baseline to 12 months between trial patients and clinical cohort patients was observed. While no difference was observed in baseline KOOS_4_ scores for patients treated with exercise therapy, other differences in patient characteristics were observed between DREAM trial patients and cohort patients.

It is not surprising that differences in patient characteristics and baseline symptom level were observed between patients receiving the same treatment in the DREAM trial and the cohort (Supporting Information S1: Tables 2 and 3). In a trial, patients are randomized to treatment, whereas treatment in daily clinical practice (i.e., the clinical cohort) is selected based on patient status, and clinician and patient preferences (e.g., a patient with acute trauma/more symptoms may have a higher chance of being recommended/select surgery), leading to risk of ‘confounding by indication’ when comparing treatment effects between DREAM trial and cohort patients [25]. Despite differences between patients randomized and selected for the different treatments, changes in patient‐reported outcomes over time were remarkably similar when comparing patients receiving the same type of treatment in the DREAM trial and the clinical cohort. While the surgical treatment in the DREAM trial and cohort can be expected to be almost identical, the exercise therapy intervention delivered in the trial followed a standardized protocol, whereas the exercise therapy delivered in daily clinical practice is likely more variable. This suggests that a specific exercise therapy protocol is not superior to a more pragmatic approach decided by the treating physiotherapist. This is supported by the Dutch STARR trial, which had a similar design and results as the DREAM trial (i.e., comparing a strategy of early meniscal surgery to a strategy of exercise therapy, with the option of later surgery for young patients with meniscal tears). In the STARR trial, an exercise protocol including guiding principles for treating physiotherapists yielded similar improvements in patient‐reported outcomes compared with meniscal surgery [24]. However, to confirm that a specific exercise therapy protocol is not superior to a more pragmatic approach, a head‐to‐head RCT comparing exercise protocols is needed.

Our study has limitations. First, only 103 patients of the 182 patients included in the baseline comparison of trial and clinical cohort patients could be included in the comparison of treatment effects, as patients either lacked information on treatment type (*n* = 71) or reported other types of treatment (*n* = 8). That said, the baseline characteristics of the 103 included and the 79 patients excluded were similar (Supporting Information S1: Table 4). Second, differential loss to follow‐up, due to the larger efforts in the DREAM trial to ensure follow‐up compared to the cohort (i.e., resulting in a higher cohort dropout rate), may have led to attrition bias, but the impact of this bias on estimates is unknown. Third, DREAM patients were included in the surgery/exercise group in our analyses according to the group to which they were originally randomized (i.e., according to the intention‐to‐treat principle). In the primary report of the DREAM trial, we also performed an ‘as‐treated’ analysis comparing all patients eventually having surgery to those not having surgery at any time up to 12 months, which confirmed the primary finding of no statistically significant difference in improvement between groups. In the present study, it was not meaningful to perform such an analysis, as no information on potential later surgery in exercise patients in the cohort was available beyond the 3‐month follow‐up. Thus, the comparison of patient‐reported outcomes for the exercise groups includes patients who may have later opted for surgery (i.e., risk of misclassification bias). Fourth, treatment in the clinical cohort was not standardized and reflected daily clinical practice and information about treatment was based on self‐report at the 3‐month follow‐up. Finally, other outcomes such as return to sport, reoperation rates or imaging outcomes are also important for this patient group. However, this type of data was not available for comparison between the two study populations.

## CONCLUSION

Baseline patient characteristics of patients enroled in the DREAM trial comparing a strategy of early meniscal surgery to a strategy of exercise therapy and patient education (with the option of later surgery) were largely comparable to the population of patients aged 18–40 years in a parallel clinical cohort, supporting that patients in the DREAM trial resemble the patients encountered in daily clinical practice. Furthermore, improvements in patient‐reported outcomes of both surgery and exercise therapy observed in the DREAM trial were comparable to the improvements observed in patients in the clinical cohort, despite some differences in baseline symptoms between patients randomized to surgery and patients selected for surgery in the cohort. The risk of potential selection bias, differential loss to follow‐up and limitations in treatment classification and data completeness in the clinical cohort should be taken into account when interpreting these findings.

## AUTHOR CONTRIBUTIONS

**Jonas Bloch Thorlund**: conceptualization, methodology, formal analysis, investigation, writing—original draft. **Dorte Thalund Grønne**: conceptualization, methodology, data curation, investigation, writing—review and editing. **Per Hölmich**: conceptualization, investigation, writing—review and editing. **Martin Lind**: conceptualization, investigation, writing—review and editing. **Søren Thorgaard Skou**: conceptualization, methodology, investigation, writing—review and editing.

## FUNDING INFORMATION

Funding information Independent Research Fund Denmark, Grant/Award Numbers: DFF‐6110‐00045, DFF‐6110‐00045B; IMK Almene Fond; Lundbeck Foundation; Spar Nord Foundation; Danish Rheumatism Association; Associationof Danish Physiotherapists Research Fund; Research Council at Næstved‐Slagelse‐Ringsted Hospitals; Region Zealand (Exercise First program grant).

## CONFLICT OF INTEREST STATEMENT

Jonas Bloch Thorlund reports a research grant from the Novo Nordisk Foundation worth 20,000 € outside the submitted work. Søren Thorgaard Skou has received personal fees from Munksgaard and TrustMe‐Ed outside the submitted work. He is one of the founders of Good Life with OsteoArthritis in Denmark (GLA:D®), which is a non‐profit initiative hosted at the University of Southern Denmark aimed at implementing clinical guidelines (exercise therapy and education) for knee and hip osteoarthritis in clinical practice. Dorte Thalund Grønne is financed by a Grant from Danish Regions (No R232‐A5132), a grant from Næstved‐Slagelse‐Ringsted Hospitals (No. A1683) and a faculty grant from the Department of Sports Science and Clinical Biomechanics, University of Southern Denmark, which are all outside the submitted work. The remaining authors declare no conflict of interest.

## ETHICS STATEMENT

The DREAM trial and cohort were approved by the Regional Committees on Health Research Ethics for Southern Denmark (S‐20160151) and the Danish Data Protection Agency (University of Southern Denmark, 16/45314). The DREAM trial was registered at ClinicalTrials.gov (NCT02995551).

## Supporting information

Supporting File

## Data Availability

The data that support the findings of this study are available on request from the corresponding author. The data are not publicly available due to privacy or ethical restrictions.
