# Supplementary material for: Comparing participants from a randomized trial and screened non‐participants: Implications for generalizability of surgery and exercise therapy in young adults with meniscal tears
Source: J Exp Orthop. 2026 May 29;13(2):e70791. doi: 10.1002/jeo2.70791 (PMC13239727; doi:10.1002/jeo2.70791)
Supplement: Supplementary file 1 — Supporting File [file JEO2-13-e70791-s001.docx]

**SUPPLEMENTARY TABLES**

**Supplementary table 1:** Comparison of mean (95% CI) Knee injury and Osteoarthritis Outcomes Scores (KOOS_4_) at all time points.

|  | **Trial** | **Cohort** | **Diff. trial vs cohort**  **(95% CI)** |
| --- | --- | --- | --- |
| **Surgery:** |  |  |  |
| Baseline | 58.6 (54.4 to 62.9) | 47.7 (43.1 to 52.3) | 10.9 (4.7 to 17.2) |
| 3 months | 73.3 (68.5 to 78.1) | 61.5 (56.9 to 66.1) | 11.8 (5.1 to 18.4) |
| 6 months | 80.1 (74.6 to 85.5) | 68.3 (62.9 to 73.8) | 11.7 (4.1 to 19.4) |
| 12 months | 78.6 (73.4 to 83.9) | 68.0 (62.0 to 74.0) | 10.6 (2.7 to 18.6) |
| **Exercise:** |  |  |  |
| Baseline | 53.1 (48.9 to 57.3) | 58.4 (53.7 to 63.1) | -5.3 (-11.6 to 1.0) |
| 3 months | 66.7 (62.2 to 71.2) | 68.1 (63.4 to 72.8) | -1.4 (-7.9 to 5.1) |
| 6 months | 69.2 (64.5 to 74.0) | 71.6 (66.3 to 76.9) | -2.3 (-9.4 to 4.8) |
| 12 months | 69.7 (64.8 to 74.5) | 71.6 (65.6 to 77.5) | -1.9 (-9.6 to 5.8) |

**Supplementary Table 2:** Comparison of baseline patient characteristics for patients having ***surgery***, DREAM trial vs. DREAM cohort.

|  | **Trial (n=60)** | **Cohort (n=52)** | **p-value** |
| --- | --- | --- | --- |
|  |  |  |  |
| Age, mean (SD) | 28.2 (6.5) | 29.2 (7.1) | 0.45 |
| Females, n (%) | 18 (30%) | 13 (25%) | 0.56 |
| BMI, mean (SD)^#^ | 25.5 (4.3) | 25.6 (4.2) | 0.93 |
| Tegner, median (IQR)^€^ | 6 (5 to 8.5) | 5 (4 to 9) | 0.28 |
| Symptom duration: |  |  |  |
| 0-3 months | 9 (15%) | 22 (44%) |  |
| 4-6 months | 25 (42%) | 13 (26%) |  |
| 7-12 months | 11 (18%) | 7 (14%) | 0.01 |
| 13-24 months | 7 (12%) | 6 (12%) |  |
| >24 months | 8 (13%) | 2 (4%) |  |
| Symptom onset: |  |  |  |
| Slowly evolved over time | 13 (22%) | 6 (12%) |  |
| Semi-traumatic | 25 (42%) | 24 (48%) | 0.41 |
| Traumatic | 22 (37%) | 20 (40%) |  |
| Positive clinical tests: |  |  |  |
| Medial joint line tenderness, n (%) | 40 (68%) | 33 (73%) | 0.54 |
| Lateral joint line tenderness, n (%) | 17 (29%) | 8 (18%) | 0.21 |
| Thessaly, n (%) | 46 (78%) | 37 (88%) | 0.19 |
| McMurray, n (%) | 42 (71%) | 38 (88%) | 0.04 |
| Mechanical symtoms, n (%) | 33 (55%) | 31 (62%) | 0.36 |
| KOOS scores, mean (SD): |  |  |  |
| KOOS_4_ | 58.6 (15.1) | 47.9 (15.8) | >0.01 |
| Pain | 69.0 (15.4) | 59.1 (18.2) | >0.01 |
| Symptoms | 69.5 (16.5) | 59.1 (17.3) | >0.01 |
| ADL | 78.3 (16.2) | 70.0 (19.9) | 0.02 |
| Sport/Rec | 46.5 (24.3) | 28.5 (21.5) | >0.01 |
| QOL | 49.6 (16.8) | 44.9 (16.1) | 0.14 |
| WOMET total %score, mean (SD) | 49.0 (20.5) | 40.6 (17.2) | 0.02 |

Percentages may not add up to 100% due to rounding. Missing data: age n=2; BMI n=3; Tegner n=6; Symptom duration n=2; Symptom onset n=2; Medial joint line tenderness n=8; Lateral joint line tenderness n=9; Thessaly n=11; McMurray n=10, Mechanical symptoms n=2; KOOS scores n=2; WOMET =2.
KOOS= Knee Injury and Osteoarthritis Outcome Score (range: 0=worst to 100=best); ADL=Function during activities of daily living; Sport/Rec=Function during sport and recreational activities; QOL=Quality of Life.
**^#^**BMI=body-mass index calculated as weight in kilograms divided by the square of the height in meters.
 **^€^**The Tegner Activity Scale ranges from 0 to 10, with 0 representing sick leave or disability pension because of knee problems to 10 representing competitive sports such as European football (national and international elite level).

**Supplementary Table 3:** Comparison of baseline patient characteristics for patients having ***exercise therapy***, DREAM trial vs. DREAM cohort.

|  | **Trial (n=61)** | **Cohort (n=51)** | **p-value** |
| --- | --- | --- | --- |
|  |  |  |  |
| Age, mean (SD) | 31.1 (6.5) | 28.7 (5.9) | 0.05 |
| Females, n (%) | 16 (26%) | 23 (45%) | 0.04 |
| BMI, mean (SD)^#^ | 26.8 (4.8) | 25.5 (4.7) | 0.15 |
| Tegner, median (IQR)^€^ | 6 (5 to 7) | 5 (5 to 8.5) | 0.96 |
| Symptom duration: |  |  |  |
| 0-3 months | 15 (25%) | 20 (41%) |  |
| 4-6 months | 20 (33%) | 14 (29%) |  |
| 7-12 months | 13 (21%) | 6 (12%) | 0.43 |
| 13-24 months | 4 (7%) | 3 (16%) |  |
| >24 months | 9 (15%) | 6 (12%) |  |
| Symptom onset: |  |  |  |
| Slowly evolved over time | 19 (31%) | 5 (10%) |  |
| Semi-traumatic | 24 (39%) | 21 (43%) | 0.02 |
| Traumatic | 18 (30%) | 23 (47%) |  |
| Positive clinical tests: |  |  |  |
| Medial joint line tenderness, n (%) | 37 (64%) | 33 (72%) | 0.39 |
| Lateral joint line tenderness, n (%) | 22 (39%) | 9 (20%) | 0.04 |
| Thessaly, n (%) | 51 (88%) | 31 (72%) | 0.04 |
| McMurray, n (%) | 41 (71%) | 29 (66%) | 0.61 |
| Mechanical symtoms, n (%) | 30 (49%) | 16 (33%) | 0.08 |
| KOOS scores, mean (SD): |  |  |  |
| KOOS_4_ | 53.1 (16.7) | 58.3 (18.0) | 0.12 |
| Pain | 63.8 (18.1) | 70.6 (17.0) | 0.04 |
| Symptoms | 68.1 (17.4) | 68.9 (19.3) | 0.83 |
| ADL | 74.7 (19.6) | 80.9 (15.8) | 0.08 |
| Sport/Rec | 38.3 (23.7) | 43.6 (28.6) | 0.29 |
| QOL | 42.1 (18.4) | 50.2 (18.8) | 0.03 |
| WOMET total %score, mean (SD) | 41.8 (18.2) | 50.2 (17.6) | 0.02 |

Percentages may not add up to 100% due to rounding. Missing data: age n=2; BMI n=2; Tegner n=6; Symptom duration n=2; Symptom onset n=2; Medial joint line tenderness n=8; Lateral joint line tenderness n=9; Thessaly n=11; McMurray n=10, Mechanical symptoms n=2; KOOS scores n=2; WOMET =2.
KOOS= Knee Injury and Osteoarthritis Outcome Score (range: 0=worst to 100=best); ADL=Function during activities of daily living; Sport/Rec=Function during sport and recreational activities; QOL=Quality of Life,
**^#^**BMI=body-mass index calculated as weight in kilograms divided by the square of the height in meters.
 **^€^**The Tegner Activity Scale ranges from 0 to 10, with 0 representing sick leave or disability pension because of knee problems to 10 representing competitive sports such as European football (national and international elite level).

**Supplementary Table 4:** Comparison of baseline patient characteristics of ***cohort*** patients included and excluded in the analyses of treatment effects.

|  | **Included (n=103)** | **Excluded (n=79)** | **p-value** |
| --- | --- | --- | --- |
|  |  |  |  |
| Age, mean (SD) | 29.0 (6.5) | 28.7 (6.4) | 0.78 |
| Females, n (%) | 36 (35%) | 28 (35%) | 0.95 |
| BMI, mean (SD)^#^ | 25.5 (4.4) | 25.9 (4.4) | 0.57 |
| Tegner, median (IQR)^€^ | 5 (4 to 9) | 6 (5 to 7) | 0.99 |
| Symptom duration: |  |  |  |
| 0-3 months | 42 (42%) | 29 (37%) |  |
| 4-6 months | 27 (27%) | 22 (28%) |  |
| 7-12 months | 13 (13%) | 12 (15%) | 0.90 |
| 13-24 months | 9 (9%) | 10 (13%) |  |
| >24 months | 8 (8%) | 6 (8%) |  |
| Symptom onset: |  |  |  |
| Slowly evolved over time | 11 (11%) | 19 (24%) |  |
| Semi-traumatic | 45 (46%) | 22 (28%) | 0.02 |
| Traumatic | 43 (43%) | 38 (48%) |  |
| Positive clinical tests: |  |  |  |
| Medial joint line tenderness, n (%) | 66 (73%) | 54 (71%) | 0.83 |
| Lateral joint line tenderness, n (%) | 17 (19%) | 29 (38%) | 0.01 |
| Thessaly, n (%) | 68 (80%) | 51 (71%) | 0.18 |
| McMurray, n (%) | 67 (77%) | 48 (67%) | 0.15 |
| Mechanical symtoms, n (%) | 47 (47%) | 37 (47%) | 0.93 |
| KOOS scores, mean (SD): |  |  |  |
| KOOS_4_ | 53.0 (17.7) | 54.5 (18.7) | 0.60 |
| Pain | 64.8 (18.4) | 64.6 (19.9) | 0.93 |
| Symptoms | 63.9 (18.9) | 64.7 (21.4) | 0.79 |
| ADL | 75.4 (18.7) | 73.8 (20.3) | 0.59 |
| Sport/Rec | 36.0 (26.3) | 42.4 (29.0) | 0.12 |
| QOL | 47.5 (17.6) | 46.2 (20.2) | 0.65 |
| WOMET total %score, mean (SD) | 45.4 (18.0) | 46.5 (20.0) | 0.68 |

Percentages may not add up to 100% due to rounding. Missing data: age n=4; BMI n=8; Tegner n=4; Symptom duration n=4; Symptom onset n=4; Medial joint line tenderness n=15; Lateral joint line tenderness n=16; Thessaly n=25; McMurray n=23, Mechanical symptoms n=4.
KOOS= Knee Injury and Osteoarthritis Outcome Score (range: 0=worst to 100=best); ADL=Function during activities of daily living; Sport/Rec=Function during sport and recreational activities; QOL=Quality of Life,
**^#^**BMI=body-mass index calculated as weight in kilograms divided by the square of the height in meters.
 **^€^**The Tegner Activity Scale ranges from 0 to 10, with 0 representing sick leave or disability pension because of knee problems to 10 representing competitive sports such as European football (national and international elite level).
